# Modeling methods for estimating HIV incidence: a mathematical review

**DOI:** 10.1186/s12976-019-0118-0

**Published:** 2020-01-22

**Authors:** Xiaodan Sun, Hiroshi Nishiura, Yanni Xiao

**Affiliations:** 1 Department of Applied Mathematics, Xi’an Jiaotong University, No 28, Xianning West Road, Xi’an, Shaanxi, 710049 China; 2 Graduate School of Medicine, Hokkaido University, Kita 15 Jo Nishi 7 Chome, Kitaku, Sapporo, 0608638 Japan

**Keywords:** statistical estimation, HIV/AIDS, CD4, Biomarker, Mathematical model

## Abstract

Estimating HIV incidence is crucial for monitoring the epidemiology of this infection, planning screening and intervention campaigns, and evaluating the effectiveness of control measures. However, owing to the long and variable period from HIV infection to the development of AIDS and the introduction of highly active antiretroviral therapy, accurate incidence estimation remains a major challenge. Numerous estimation methods have been proposed in epidemiological modeling studies, and here we review commonly-used methods for estimation of HIV incidence. We review the essential data required for estimation along with the advantages and disadvantages, mathematical structures and likelihood derivations of these methods. The methods include the classical back-calculation method, the method based on CD4+ T-cell depletion, the use of HIV case reporting data, the use of cohort study data, the use of serial or cross-sectional prevalence data, and biomarker approach. By outlining the mechanistic features of each method, we provide guidance for planning incidence estimation efforts, which may depend on national or regional factors as well as the availability of epidemiological or laboratory datasets.

## Background

Since the first patient with acquired immunodeficiency syndrome (AIDS) was reported in 1981 [1], its causative agent, human immunodeficiency virus (HIV), has led to 77 million HIV infections globally and remains a major public health issue [2]. To strategically assess the impact of interventions and to guide policy makers in achieving improved control of HIV/AIDS, it is critical to quantify the dynamics of HIV epidemics accurately and reliably. HIV incidence (i.e., the transient number of new infections) and prevalence (i.e., the fraction of infected individuals at a given point in time) are two major indicators that are used to assess and interpret the transmission dynamics of HIV. HIV incidence and prevalence have been estimated using mathematical and statistical modeling approaches by many academic and governmental research groups. For instance, the Joint United Nations Program on HIV/AIDS (UNAIDS) regularly provides updates of national and global estimates, indicating that 1.8 million people were newly infected with HIV and 940,000 deaths occurred in the year 2017 [2].

Unlike many acute infectious diseases, HIV infection progresses slowly in vivo and has a complex natural history. During the first 2-4 weeks following infection, the virus replicates rapidly and this period is referred to as the acute stage [3, 4]. Thereafter, viral loads are greatly reduced and reach a quasi-steady state, which is called the asymptomatic stage. During the asymptomatic stage, the viral load reflects the steady state achieved between high rates of viral replication and virus clearance, and is maintained at a remarkably stable level (i.e., the viral load set point) over a number of years. If untreated, the median length of asymptomatic stage may range from 8-11 years. Infected individuals in the asymptomatic stage do not show overt symptoms but can transmit HIV infection through high-risk behaviors. Subsequently, the viral load increases slowly, resulting in the onset of AIDS [5–7]. Because their immune systems are severely damaged, individuals with AIDS experience a number of opportunistic infections and are at high risk of death without treatment.

Owing to the lengthy asymptomatic stage without symptoms, many individuals do not realize that they are infected for a number of years. Moreover, through sexual contact and intravenous drug use, infections often remain undetected due to the reliance on voluntary testing following those high risk exposures[8, 9]. This issue both leads to increased HIV transmission and complicates modeling exercises, increasing the difficulty of explicitly quantifying the epidemiological dynamics of HIV/AIDS. Furthermore, owing to the widespread use of antiretroviral therapy (ART), prevalence estimation is controversial: even where prevalence can be estimated, this estimate may not reflect the current dynamics of HIV epidemics and may reflect only the degree of spread from many years in the past [10]. It is generally recognized that estimation of HIV incidence can provide greater insights into the real-time evaluation of HIV epidemics. Nevertheless, the long asymptomatic stage also causes challenges in estimating HIV incidence.

Starting in the 1980s, a large number of modeling studies have aimed to estimate HIV incidence, and a variety of useful methods have been proposed for this purpose. These diverse methods have played important roles in HIV incidence estimation in different parts of the world. However, only brief comparative notes have been published elsewhere [10–12], aiming for improvement in practical estimation settings. In this review, we comprehensively describe the major methods that have been used for HIV incidence estimation, including (i) the classical back-calculation method, (ii) the method based on CD4+ T-cell depletion, (iii) the use of HIV case reporting data, (iv) the use of cohort study data, (v) the use of serial or cross-sectional prevalence data, and (vi) biomarker approach. We focus on the structural mechanisms of modeling as well as the mathematical derivation of likelihood functions, and compare the advantages and disadvantages of existing methods. Our review is targeted to a general audience in theoretical biology. Finally, we summarize important implications for future development of estimation methods for HIV incidence.

## Back-calculation

Back-calculation, one of the most widely-used statistical modeling approaches, exploits the distribution of incubation periods of AIDS. The back-calculation method uses epidemiological surveillance data to reconstruct HIV infections over time. The basic idea of the method can be described as follows. If the rate of incident HIV infections at time *s* is *I*(*s*), and the probability density function of the incubation period *f*(*s*) is known, then AIDS incidence at time *t*, denoted by *A*(*t*), can be described by

1 $$\small A(t)=\int_{0}^{t} I(t-\tau)f(\tau)d\tau.  $$

Conversely, if the dataset for *A*(*t*) is available from surveillance data and *f*(*s*) can be determined from the literature, HIV incidence can be “back-calculated” by rearranging (1) to

2 $$\small A(t)=\int_{0}^{t} I(s)f(t-s)ds.  $$

If *F*(*t*) denotes the cumulative distribution function of the incubation period, one can describe the expected number of AIDS diagnoses over the time interval [*T*_*i*−1_,*T*_*i*_], denoted by *X*_*i*_, as

3 $$ {{}\begin{aligned} \begin{array}{rcl}\small E(X_{i}) &=& \int_{T_{i-1}}^{T_{i}} \int_{0}^{t} I(s)f(t-s)ds dt \\ &=&\int_{0}^{T_{i-1}}\int_{T_{i-1}}^{T_{i}} I(s)f(t-s)dt ds \\&+&\int_{T_{i-1}}^{T_{i}}\int_{s}^{T_{i}}I(s)f(t-s)dt ds\\ &=& \int_{0}^{T_{i-1}}I(s) \int_{T_{i-1}}^{T_{i}} f(t-s)dt ds \\&+& \int_{T_{i-1}}^{T_{i}} I(s)\int_{s}^{T_{i}}f(t-s)dt ds\\ &=&\int_{0}^{T_{i-1}}I(s)[F(T_{i}-s)-F(T_{i-1}-s)] ds \\ &&+ \int_{T_{i-1}}^{T_{i}} I(s) [F(T_{i}-s)-F(0)] ds \\ &=& \int_{0}^{T_{i}} I(s)[F(T_{i}-s)-F(T_{i-1}-s)] ds. \end{array} \end{aligned}}  $$

Here, the last equality holds because *F*(*T*_*i*−1_−*s*)=*F*(0)=0 for *T*_*i*−1_−*s*≤0. Then, we have

4 $$\small E(X_{i}) = \int_{0}^{T_{i}} I(s)[F(T_{i}-s)-F(T_{i-1}-s)] ds.  $$

### The classic method using AIDS data

The back-calculation method was first proposed by Brookmeyer et al. [13–15] who used AIDS incidence data to estimate discrete HIV incidence using the maximum likelihood estimation method. Let *T*_0_,*T*_1_,⋯,*T*_*L*_ denote discrete times, *N* denote the total number of infections before *T*_*L*_, and *X*_*i*_ denote the number of diagnosed AIDS cases in the *i*th time interval [*T*_*i*−1_,*T*_*i*_]. Then, *N* is the sum of all infected cases that have been diagnosed, $X.=\sum _{i=1}^{L} X_{i}$, and those infected before *T*_*L*_ but have not been diagnosed are indicated by *X*_*L*+1_=*N*−*X*.. Suppose that *X*=(*X*_1_,*X*_2_,⋯,*X*_*L*_,*X*_*L*+1_) follows a multinomial distribution with sample size *N*, where probabilities (*p*_1_,*p*_2_,⋯,*p*_*L*_,1−*p*_._) can be calculated according to Eq. (3), and $p_.= \sum _{j=1}^{L} p_{j}$. In fact, $p_{i}=\int _{T_{0}}^{T_{i}} i(s)[F(T_{i}-s)-F(T_{i-1}-s)] ds$, where *i*(*s*) is the probability density function for these *N* individuals being infected at time *s*. Denoting the observed AIDS incidence in each time interval as *x*_1_,*x*_2_,⋯,*x*_*L*_, the likelihood function can be described as follows:

$${{}\begin{aligned} \frac{N!}{x_{1}!x_{2}!\cdots x_{L}! \left(N\,-\,\sum_{i=1}^{L}x_{i}\right)!}p_{1}^{x_{1}}p_{2}^{x_{2}}\!\cdots\! p_{L}^{x_{L}}(1\,-\,p_.)^{N-\sum_{i=1}^{L}x_{i}}. \end{aligned}} $$

The back-calculation method can estimate the historical incidence of infection that was already diagnosed and also the number of infections that have yet to be diagnosed.

Becker et al. [16] proposed a non-parametric approach to this method using the discrete version of Eq. (2). Let the number of HIV infections in the *i*th time interval be *I*_*i*_ and the probability mass function of the incubation period be *f*_*d*_. Then, the expected number of AIDS diagnoses in interval *i* can be described as

5 $$\small E(X_{i}|I_{1},I_{2},\cdots,I_{i})=\sum_{j=1}^{i} I_{j} f_{i-j}.  $$

Let *μ*_*i*_=*E*(*X*_*i*_) and *λ*_*j*_=*E*(*I*_*j*_). Then,

$$\mu_{i}=\sum_{j=1}^{i}\lambda_{j} f_{i-j}.$$

Assuming that HIV infections are generated by a non-homogeneous Poisson process, *X*_*i*_ (*i*=1,⋯,*L*) would follow Poisson distributions with means *μ*_*i*_. Then, the likelihood function is

6 $$\small \prod_{i=1}^{L}\left(\sum_{j=1}^{i}\lambda_{j} f_{i-j}\right)^{x_{i}}\exp\left(-\sum_{j=1}^{i}\lambda_{j} f_{i-j}\right),  $$

where *x*_*i*_ is the observed frequency of AIDS cases.

It should be noted that this method assumes that the distribution of the incubation period does not vary over time. In fact, it is easy to modify Eq. (2) to $A(t)=\int _{0}^{t} I(s)f(t-s\mid s)ds$, where *f*(*t*−*s*∣*s*) is the probability density function for an individual who was infected at time *s* and diagnosed at time *t*. Thus, *f*(*t*−*s*∣*s*) describes the time-dependent distribution of incubation period. Similarly, the discrete version of Eq. (5) becomes $E(X_{i}|I_{1},I_{2},\cdots,I_{i})=\sum _{j=1}^{i} I_{j} f_{i-j, j}$ with *f*_*i*−*j*,*j*_ representing the probability for an individual infected during time interval [*t*_*j*−1_,*t*_*j*_) and diagnosed during time interval [*t*_*i*−1_,*t*_*i*_).

In this way, the mathematical expression of the back-calculation method is straightforward, but the estimation of *I*_*i*_ using this method is challenging [17] because of the high dimension of *I*_*i*_ which leads to instability. To estimate the incidence of HIV *I*_*i*_, several published studies [18, 19] have used either *A*(*t*) or *I*(*t*) as flexible parametric functions. Rosenberg et al. [20] estimated the infection curve *I*(*s*) directly, assuming that *I*(*s*) is a member of the general family *G*={*g*_1_(*s*),⋯,*g*_*I*_(*s*)}, where *g*_*i*_(*s*) are integrable real functions. That is,

$$I(s)=\Sigma_{i=1}^{I}g_{i}(s)\beta_{i}.$$

Specifically, this method includes splines and step functions.

It should be noted that for models involving spline and step functions, another weakness is the potential of overfitting and the ill-posed inverse problem. Overfitting arises when too many knots in the spline are applied. The ill-posed problem arises when the step function is too discrete and when the estimated HIV incidence becomes overly sensitive to temporal fluctuations of data points. Moreover, when the HIV epidemic has just started and the trend has not been stable, the back-calculated incidence in the most recent years would be more uncertain than that based on long-lasting epidemic dynamics. This is caused by small number of diagnosed infections in recent observed times, yielding substantial uncertainties. However, in many existing settings in developed countries, the HIV epidemic has continued for substantial number of years, and in such an occasion, the uncertainties in the estimated recent infections are not as large as that estimated in the early epidemic phase with dramatic peaks and troughs, as shown by Yan and Zhang [21]. In addition, this method has been criticized because the estimation strongly depends on the distribution of the incubation period, which needs to be determined from other cohorts. Estimation of the incubation period encountered critical challenges in the 1990s, as the introduction of ART extended the length of the incubation period, inevitably changing this distribution. To account for the effect of ART, extended methods were proposed [22–26].

### Using both HIV and AIDS diagnoses

In addition to AIDS incidence, the frequency of diagnosed HIV infections has become available as part of epidemiological surveillance data, greatly assisting researchers to extend the back-calculation method [27–40]. Early studies used only HIV diagnoses of individuals who later progressed to AIDS [27–32]. Subsequently, several other methods were proposed to incorporate all HIV diagnoses, including infected individuals who have not yet developed AIDS [33–40]. Yan et al. [41] proposed an approach which uses the number of new HIV diagnoses to back-calculate historical HIV incidence, partially aided by supplementary data from the old AIDS case surveillance system in populations where there were such system in the 1980s. The estimate is also calibrated with supplementary data based on “recent infections”, that is, the proportion among newly diagnosed HIV that is recently infected according to enhanced surveillance or laboratory assays. This method was used to estimate HIV incidence among men who have sex with men in Australia [42, 43]. Adding information on HIV diagnoses to the back-calculation method enables estimation of HIV incidence in recent years and reduces the uncertainty associated with this estimate to some degree. Moreover, the method enables joint estimation of HIV diagnosis rate [44]. However, challenges associated with estimating or assuming a time from infection to HIV diagnosis remain.

### Including CD4+ T-cell counts at diagnosis

Upon HIV diagnosis, CD4+ T-cell count data has now become widely available. Various studies have defined HIV/AIDS progression based on CD4+ T-cell counts and employed Markov process models [33, 38] to estimate HIV incidence. Birrell et al. [45] formulated a CD4-stage structured model to use CD4+ T-cell counts at diagnosis. CD4+ T-cell count data represented the first CD4 count recorded within three months of HIV diagnosis. The model included a total of five stages: CD4+ T-cell counts of [500,*∞*),[350,500),[250,300),[0,200), and the AIDS stage. Infected individuals are assumed to experience progressive decline in CD4+ T-cell counts and proceed through the five stages before they are diagnosed with AIDS. Let *d*_*j*_=(*d*_1,*j*_,*d*_2,*j*_,*d*_3,*j*_,*d*_4,*j*_), and *d*_*k*,*j*_,*k*=1,2,3,4 denote the probability of diagnosis in the *k*th CD4 count stage during time interval *j*. Let *e*_*j*_=(*e*_1,*j*_,*e*_2,*j*_,*e*_3,*j*_,*e*_4,*j*_), where *e*_*k*,*j*_,*k*=1,2,3,4 denotes the expected number of undiagnosed infections in the *k*th CD4 count stage during time interval *j*. Suppose that the expected number of diagnosed HIV and AIDS cases during time interval *j* is $\mu _{j}^{HIV} and \mu _{j}^{AIDS}$, respectively, then

7 $$ \begin{array}{rcl} \mu_{j}^{HIV} &=& \mathbf{e_{j-1}}\cdot \mathbf{d_{j}}^{T}, and\\ \mu_{j}^{AIDS} &=& e_{4, j}(1-d_{4, j})\rho_{4,5} \end{array}  $$

where

$$\mathbf{e_{j}}=\mathbf{P_{j}^{T}e_{j-1}}+(\lambda_{j},0,0,0)^{T}.$$

*λ*_*j*_ is the expected number of new HIV infections in time interval *j*, and *P*_*j*_ is the transition matrix describing the proportion of individuals transitting between different stages during time interval *j*. Then,

8 $$\small (\mathbf{P_{j}})_{k,l}=\left\{ \begin{array}{lcl} (1-d_{k,j})(1-\rho_{k,k+1}) &\quad& k=l, \\ (1-d_{k, j})\rho_{k,k+1} &\quad& k=l-1, \\ 0 & \quad & \text{otherwise.} \end{array} \right.  $$

where *ρ*_*k*,*k*+1_ is the transition probability from stage *k* to *k*+1. Let *X*_*j*_ and *Y*_*j*_(*j*=1,⋯,*L*) denote AIDS and HIV diagnoses during the time interval *j*, respectively, which are assumed to follow independent Poisson distributions with means $\mu _{j}^{HIV}$ and $\mu _{j}^{AIDS}$, respectively. Then, the likelihood function for HIV and AIDS diagnoses can be calculated as

$${{}\begin{aligned} L_{1}(\mathbf{X,Y;h,d})&\propto \prod_{j=1}^{L} \left(\mu_{j}^{AIDS}\right)^{X_{j}}\\&\exp\left(-\mu_{j}^{AIDS}\right)\times \left(\mu_{j}^{HIV}\right)^{Y_{j}}\exp\left(-\mu_{j}^{HIV}\right). \end{aligned}} $$

CD4+ T-cell count data at diagnosis is also available for a subset of the above-diagnosed HIV-positive individuals. The CD4+ T-cell count data at diagnosis are divided into four sets: [500,*∞*),[350,500),[250,300), and [0,200). Let *C*_*j*_=(*C*_1,*j*_,*C*_2,*j*_,*C*_3,*j*_,*C*_4,*j*_) or *C*_*k*,*j*_(*k*=1,2,3,4) be the number of HIV-positive individuals whose CD4 counts fall into the *k*th CD4 stage during the *j*th time interval, and $N_{j}=\Sigma _{k=1}^{4} C_{k,j}$. That is, *N*_*j*_ individuals are diagnosed with HIV during the time interval *j* with the state variable, CD4-at-diagnosis data. We assume that these *N*_*j*_ HIV-positive individuals with CD4 data are multinomially distributed as

$$\mathbf{C_{j}} \sim Multinomial (N_{j}, \mathbf{r_{j}}),$$

where

$$\mathbf{r_{j}}=\{r_{k,j}: k=1,2,3,4\}, r_{k,j}\,=\,\frac{e_{k,j-1}d_{k,j}}{\mu_{j}^{HIV}}, j\,=\,1,2,\cdots,L.$$

Then, the likelihood of observing CD4-at-diagnosis data can be given as

$$L_{2}(\mathbf{C|D;h,d})\propto \prod_{j=1}^{L} \prod_{k=1}^{4} r_{k,j}^{C_{k,j}}.$$

The full likelihood is the product of *L*_1_ and *L*_2_:

$$L(\mathbf{X,Y,C;h,d})=L_{1}(\mathbf{X,Y;h,d})L_{2}(\mathbf{C|D;h,d}).$$

This method can make full use of all the available data, including HIV and AIDS diagnoses as well as CD4+ T-cell counts at diagnosis. Using this method, one cannot only estimate the incidence of HIV infections but also the diagnosis rates at different CD4 stages and during different time intervals, providing insightful information to comprehensively evaluate the epidemiology of HIV/AIDS. Using this model, Birrell et al. estimated HIV incidence in England and Wales [46], and found that the mean time to diagnosis had shortened from 2001 to 2010 owing to expansion of HIV testing. However, this method is also highly dependent on the progression rate between different stages. Moreover, the quantities requiring estimation have much higher dimensions yielding additional difficulties. Birrell et al. [45] employed the Bayesian estimation technique, ensuring the stability of estimates.

## Using CD4+ T-cell count data at diagnosis based on a CD4+ T-cell depletion model

In addition to the back-calculation method, another major HIV incidence estimation method is to jointly use HIV diagnosis data and the first CD4 count data while employing the CD4+ T-cell depletion model [47–49]. This method first estimates the distribution of diagnosis delays (i.e., the time from infection to diagnosis), and then estimates the incidence of HIV from the depletion of CD4+ T-cells [49]. Here, HIV incidence refers to the number of new infections during each time interval, including both diagnosed and undiagnosed infections by the end of the study period. The CD4+ T-cell depletion model that was adopted by Lodi et al. and Touloumi et al. [50, 51] can be expressed as

$$\sqrt{CD4(t)}=a_{i}+(b_{i}\times t)+e_{it},$$

where *t* denotes the time from infection to the date of the first CD4+ T-cell count determination. Then, the time from date of infection to CD4 testing for an individual *i* can be estimated by

$$T_{i}=\frac{\sqrt{\text{first} CD4}-a_{i}}{b_{i}}.$$

*a*_*i*_ and *b*_*i*_ are assumed to follow a bivariate normal distribution *N*[(*a*,*b*),(*σ*_*a*_,*σ*_*b*_),*ρ*], and are variable from person to person. Using standard survival analysis techniques, the diagnosis delay probability *P*(*x*) was estimated, which is the probability that an infected person would be diagnosed within *x* time units after infection. To statistically estimate undiagnosed infections, the authors further defined the diagnosis delay weight as *W*(*x*)=1/*P*(*x*).

Let *t*_0_ and *t*_*N*_ be the start and end times of the study period. The estimated infection time for each diagnosed individual may be either before or after *t*_0_. Suppose the estimated number of infections in the *i*th year after *t*_0_ is *n*_*i*_ (using the CD4+ T-cell depletion model), where *i*=1,2,⋯,*N*, and the time of infection for each case is *DI*_*j*_,*j*=1,2,⋯,*n*_*i*_. Then, the number of new infections in the *i*th year after *t*_0_ can be estimated as

$$\lambda_{i}=\sum_{j=1}^{n_{i}} W(t_{N}-{DI}_{j}). $$

A certain number of individuals remain to be infected but are not diagnosed before *t*_0_. Let *U* denote the number of such individuals. These individuals may either be diagnosed between *t*_0_ and *t*_*N*_, or not diagnosed until the end of the study period *t*_*N*_. Let *u*_*i*_,*i*=1,2,⋯, be the number of newly diagnosed cases among these persons in the *i*th year after *t*_0_. Then, $U=\sum _{i\geq 1}u_{i}$ is the total number of diagnoses observed during the study period. In addition, *u*_*i*_ for *i*>*N* are cases who are not diagnosed until the end of the study period. *H*_*i*_ is further defined as the total number of cases diagnosed during the *i*th year after *t*_0_ (including those infected before and after *t*_0_), where *i*=1,⋯,*N*. Thus, *r*_*i*_=*u*_*i*_/*H*_*i*_ is the proportion of new diagnosed cases in the *i*th year after *t*_0_ who are infected before *t*_0_. Both *H*_*i*_ and *r*_*i*_ are treated as linear regression functions of time *t*, so *H*_*i*_ and *r*_*i*_ for *i*>*N* can then be predicted, and *u*_*i*_ for *i*>*N* can at last be calculated as *u*_*i*_=*H*_*i*_×*r*_*i*_. For persons who are infected but not diagnosed before *t*_0_, another diagnosis delay weight is defined as $W=U/\sum _{i=1}^{N} u_{i}$. Suppose the estimated number of infections in the *i*th year before *t*_0_ is *m*_*i*_ (using the CD4 depletion model). Then, the number of new infections in the *i*th year before *t*_0_ can be estimated as

$$\nu_{i}=m_{i} W.$$

In fact, the method based on a CD4+ T-cell depletion model is also a kind of back-calculation method (it is sometimes referred to as the extended back-calculation method) because it also uses HIV/AIDS or CD4 T-cell counts at diagnosis to ‘back-calculate’ the time of infection among infected individuals. In the classical back-calculation method, only the total number of HIV/AIDS cases is required. In the extended back-calculation method, CD4+ T-cell counts at diagnosis are required at the individual level. For non-experts, the extended back-calculation method is easier to carry out owing to its low computational complexity compared with the classical back-calculation method. Nevertheless, similar to the classical back-calculation method, the validity of the extended back-calculation method is highly dependent on the CD4+ T-cell depletion model. In many countries and geographic areas, the empirical data required to estimate parameters of the CD4+ T-cell depletion model are extremely scarce. In China for example, after the test-and-treat policy became widespread, it became much more difficult to empirically observe CD4+ T-cell count data during natural infection in the absence of ART.

## Simple method using HIV case reporting data

In 2017, Xia et al. [52] proposed a very simple novel method by which even non-experts can estimate HIV incidence using HIV case reporting data. The method assumes that HIV incidence and case finding are stable within each 3-year period. The timeframe of interest is broken down into overlapping 3-year periods (e.g., 2002−2004,2003−2005,⋯,2008−2010). The HIV incidence for the second year of each 3-year period can be estimated by solving the following equations:

$$R=\frac{D_{1}}{U_{1}+I_{1}}=\frac{D_{2}}{U_{2}+I_{2}}=\frac{D_{3}}{U_{3}+I_{3}},$$

and

$$U_{2}=U_{1}+I_{1}-D_{1},$$

$$U_{3}=U_{2}+I_{2}-D_{2},$$

$$I_{2}=I_{1}+\varepsilon_{1}, \text{ where}\ \varepsilon_{1} \text{ is small},$$

$$I_{3}=I_{2}+\varepsilon_{2}, \text{ where}\ \varepsilon_{2} \text{ is small}.$$

*R* is the case finding rate in a year, *D*_*i*_ is the number of new diagnoses in year *i*, *U*_*i*_ is the number of undiagnosed cases at the beginning of year *i*, and *I*_1_ is the HIV incidence in year *i* (*i*=1,2,3). Then,

$$I_{2}\approx\frac{D_{1}D_{3}-D_{2}D_{2}}{D_{1}-2D_{2}+D_{3}}.$$

This method is simple enough for non-experts. Moreover, it is very easy to carry out, requiring only HIV case reporting data. However, the method is applicable only if both incidence and diagnosis rates are stable over three years.

## Cohort studies

Another strategy for estimation of HIV incidence is to use cohort studies of uninfected individuals [53]. Since it is difficult to follow sufficient individuals at the national level, a cohort study design is employed for estimating incidence among subpopulations [11]. This method enables researchers to directly measure HIV incidence in the sample population, but biases are introduced when estimating incidence by cohort. These biases are mainly caused by two sources of error [11]. First, individuals who receive follow-up visits may not be representative of the population. Second, individuals who adhere to the follow-up visits may obtain counseling repeatedly, and thus, their knowledge of HIV may improve over time which could affect risk of acquiring HIV.

## Prevalence data

Incidence and prevalence are two important metrics for evaluating HIV epidemics. In fact, these two measures are related to one another. Two different types of prevalence data have been used to estimate HIV incidence: serial prevalence and cross-sectional prevalence [54–59]. In this section, we review two different incidence estimation methods using serial and cross-sectional prevalence data.

### Estimating incidence from serial prevalence surveys

UNAIDS has developed an Estimation and Projection Package (EPP) which can be used to obtain HIV prevalence and projections [57]. Another software program, SPECTRUM [58], internally linked with EPP, can be employed to calculate the HIV incidence using the AIDS Impact Model (AIM) module. Here, we summarize the simplified methodology implemented in SPECTRUM. Let *H*_*a*,*t*_,*A*_*a*,*t*_ and *P*_*a*,*t*_ denote the number of HIV infections, the total number of adults in the population and HIV prevalence of individuals aged *a* at time *t*, respectively. Thus,

$$H_{a,t}=A_{a,t}\times P_{a,t}.$$

New HIV infections (*I*_*a*,*t*_) are the number of HIV infections in year *t* minus the number of survived infections from year *t*−1. The number of survived infections from year *t*−1 can be further calculated as the number of HIV infections in year *t*−1 minus deaths among HIV infected individuals (including deaths caused by AIDS, $D^{A}_{a,t}$, or deaths from other reasons, $D^{NA}_{a,t}$) in year *t*−1:

$$I_{a,t} =H_{a,t}-\left(H_{a-1,t-1}-D^{A}_{a-1,t-1}-D^{NA}_{a-1,t-1}\right). $$

AIDS deaths in year *t* are calculated as the convolution of the number of new infections in year *t*−*i* and the proportion of deaths caused by AIDS *i* years after infection (*r*_*i*_):

$$D^{A}_{a,t-1} =\sum_{i=0}^{20}(I_{a-i,t-i}\times r_{i}).$$

SPECTRUM has been updated several times since its initial 2004 release [60–63], and the last update took place in 2017 [64]. Other studies using the similar modeling approach have been conducted to estimate HIV epidemic [65, 66]. Hallett et al. [10] indicated that this method can estimate HIV incidence from the earliest stages of the epidemic, which is helpful to evaluate HIV epidemics over time. However, if large amounts of data are available, the estimate will involve a large uncertainty as the variation range of the incidence curve is very large. Since SPECTURM need to use EPP to generate the prevalence estimate and projections, and subsequently estimate the incidence of new HIV infections, any change in the incidence can only be detected through prevalence changes that may be observed over several years in later time. An additional disadvantage of this method is the difficulty in choosing an appropriate dataset from which prevalence is estimated. The estimation of HIV incidence could be significantly biased if the prevalence for the entire population is not estimated properly. For the long time, epidemiologists have used the data from antenatal clinics to estimate the prevalence in the entire population in sub-Saharan Africa [57]. As the HIV prevalence then appeared to be greater than that of the general population, national population-based household HIV surveys data are additionally used to calibrate overall population prevalence [67–69]. In fact, EPP began to include such household survey data in the estimation [70, 71]. Besides, household survey could miss a large part of the population that was affected by the HIV epidemic, and may on the other hand yield substantially small estimate of the prevalence. Synthesizing the use of different datasets over time could act as a cause of biased estimation.

### Calculating incidence from cross-sectional prevalence

Hallett et al. [59] proposed a method to estimate the age-specific incidence of HIV from cross-sectional prevalence data. They first estimated incidence based on cohort mortality rates of infected individuals as well as survival distributions following HIV infection, then calculated age-specific incidence according to the relationship between these two measures.

In the following, we first summarize the relationship between age-specific incidence and cohort incidence. The age group *i* is defined as individuals aged from $a_{i}-\frac {r}{2}$ to $a_{i}+\frac {r}{2}$. Thus, the age group *i* is centered at *a*_*i*_ with a width of *r* years. The total number of individuals and HIV-infected individuals in age group *i* at time *j* are denoted by *N*_*i*,*j*_ and *H*_*i*,*j*_, respectively. Then, the prevalence is *p*_*i*,*j*_=*H*_*i*,*j*_/*N*_*i*,*j*_.

**Fig. 1:**
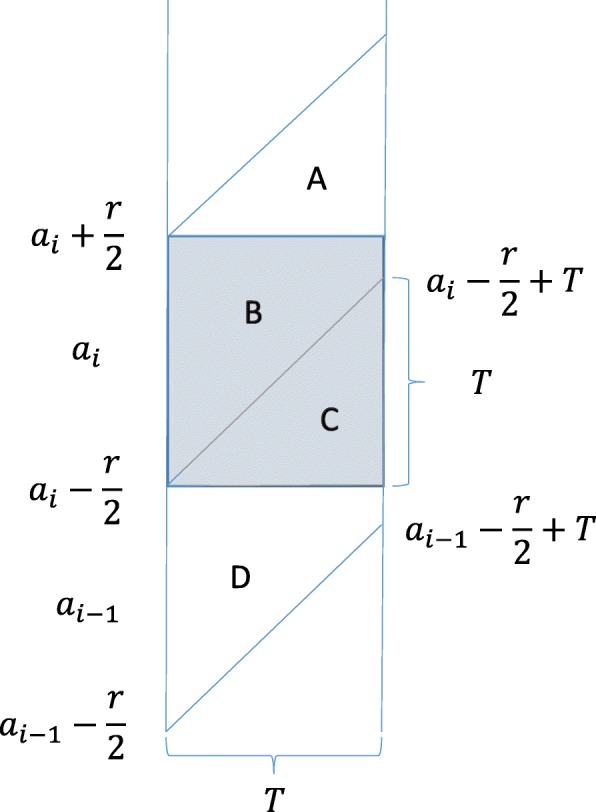
Diagram of age cohort experience of incidence and conventional age-specific incidence

We assume that cross-sectional prevalence is measured with an interval of *T* years in such age groups. Thus, age cohorts can be constructed as aged $a_{i}-\frac {r}{2}$ to $a_{i}+\frac {r}{2}$ at the start and $a_{i}-\frac {r}{2}+T$ to $a_{i}+\frac {r}{2}+T$ at the end of each interval. Now the cohort incidence, which is denoted by $\tilde {\lambda }_{i}$, can be illustrated by diagonal parallelogram (regions *A* and *B* in Fig. 1). The conventional age-specific incidence rate for age-group *i*, which is denoted by *λ*_*i*_, is illustrated by regions *B* and *C* in Fig. 1. As Fig. 1 shows, region *C* can be seen as part of the incidence of cohort *i*−1. Denote the areas of regions *A* and *B* as *S*_*A*_,*S*_*B*_. The total area for the diagonal parallelogram is *Tr* (that is, *S*_*A*_+*S*_*B*_). The fractions contributed by cohort *i* and *i*−1 are 1−*T*/2*r* (*S*_*B*_/(*S*_*A*_+*S*_*B*_)) and *T*/2*r* (*S*_*A*_/(*S*_*A*_+*S*_*B*_)), respectively. Then, the conventional age-specific incidence rate can be calculated using the following equation:

$$\lambda_{i}=\left(1-\frac{T}{2r}\right)\tilde{\lambda}_{i}+\left(\frac{T}{2r}\right)\tilde{\lambda}_{i-1}.$$

The derivation of the above formula assumes that *T*≤*r*. When *T*>*r*, a similar method can be used for deriving a different formula, which is omitted here.

In the following, the methodological background of the cohort incidence estimation $\tilde {\lambda }_{i}$ is described. Let $\tilde {\pi }_{i}$ be the fraction of infected individuals in the *i*th age-group who survive from the start to the end of the interval, and $\tilde {\mu }_{i}$ be the mortality rate during this interval for individuals in the *i*th age-group who are uninfected. Then, the number of seroconverting individuals in age group *i* during the interval *T* can be approximated as

$$H_{i,T}-\tilde{\pi}_{i} H_{i,0},$$

and the number of person-years spent by age-group *i* during the interval *T* is approximated as

$$T\frac{(N_{i,0}-H_{i,0})+(N_{i,T}-H_{i,T})}{2}.$$

Then, the cohort incidence can be derived as

$$\tilde{\lambda}_{i}=\frac{\text{seroconversions}}{\text{person-years}} =\frac{2(Q_{i}p_{i,T}-\tilde{\pi}_{i}p_{i,0})}{T(1-p_{i,0}+Q_{j}(1-p_{i,T}))},$$

where *Q*_*j*_ denotes the change in the size of the cohort over the time interval *T*.

$$Q_{j}\approx1-(1-\tilde{\pi}_{i})p_{i,0}-(1-exp(-\tilde{\mu}_{i}T))(1-p_{i,0}).$$

The authors further defined age cohort 0, calculating the prevalence at the start and end of the interval, and subsequently, the cohort incidence for this age cohort. $\tilde {\pi }_{i}$ can either be estimated based on the age-specific cohort mortality rates of infected individuals, or estimated using the distribution of survival time after infection, although we omit the details in this review. To use this method, age-specific cross-sectional prevalence data are required. Moreover, the duration between two cross-sectional measurements of prevalence should be small to ensure that the incidence and prevalence do not change significantly during this time interval. Because people with long survival time are preferably included in cohorts, the time-length bias is inevitable with this method. Both methods using serial and cross-sectional prevalence could be affected by the increasing coverage of ART [10, 59].

## Biomarker approach for cross-sectional incidence estimation

It is widely recognized that recent infection rates are difficult to estimate using the back-calculation method owing to the long incubation period of AIDS, while cohort studies have difficulty following a sufficient number of high-risk uninfected persons. To complicate these issues, ART can considerably extend the incubation period, adding complexity to the majority of estimation methods mentioned above. As a possible alternative, a biomarker-based approach using cross-sectional incidence estimation was proposed and has clear advantages in estimation of recent infections [72–86]. This approach uses biomarkers from biological samples collected in cross-sectional studies to identify recent HIV infections.

### Using diagnostic tests for the p24 antigen during the pre-seroconversion period.

In 1995, Brookmeyer et al. [72] proposed a simplistic modeling approach that uses diagnostic tests for HIV-1 p24 antigen to determine the prevalence of individuals who are p24 antigen-positive among HIV-seronegative individuals. Let *μ* be the mean duration of the p24 antigen-positive period before seroconversion, *I* be the infection risk per unit time for each uninfected individual (that is, the current incidence rate), and *p* be the expected proportion of individuals who are p24 antigen-positive among individuals whose HIV-antibody test results are negative or indeterminate. Then, *p* can be approximated as *Iμ*, and *I* can be estimated as

$$I=p/\mu.$$

Here, *μ* is referred to as the window period during which infected individuals have not yet seroconverted, but are still identifiable using biomarker(s). Supposing that $\tilde {p}$ is the number of individuals who are p24 antigen-positive during the window period, and *n* is the total number of individuals in the cross-sectional survey whose HIV antibody tests results are negative or indeterminate (i.e., $p=\frac {\tilde {p}}{n}$). Then, we have

$$I=\frac{1}{\mu}\cdot \frac{\tilde{p}}{n}.$$

The confidence interval for the incidence rate can be further estimated by assuming that $\tilde {p}$ follows a Poisson distribution with expectation *nIμ*.

### Using HIV enzyme immunoassay (EIA), antibody avidity index or genetic diversity

For the method proposed by Brookmeyer et al. [72], all individuals whose HIV antibody tests are negative need to undertake diagnostic testing for p24 antigen. Since the duration of the p24 antigen-positive pre-seroconversion period (window period) *μ* is very short (mean duration 22.5 days [72]), a large number of individuals need to be tested in situations where *I* (the population incidence rate) is high or *n* (the number of individuals that can be tested) is large. Janssen et al. [73] developed a new method to employ a testing algorithm based on either a sensitive assay (3A11) or a less-sensitive assay (3A11-LS). For a given cohort study, let *T* be the mean duration between seroconversion for the two assays (i.e., the window period), *n* be the number of individuals who are 3A11 reactive and 3A11-LS non-reactive, and *N* be the number of individuals who are HIV-negative or 3A11 reactive/3A11-LS non-reactive. Then, the incidence rate is

$$I=\frac{n}{N}\cdot\frac{1}{T}.$$

The window period using the sensitive/less sensitive assay testing algorithm is longer (i.e., 129 days) [73]. However, the algorithm does not perform well in populations infected with non-B HIV-1 subtypes [74]. Parekh et al. [75] proposed a subtype-independent assay called BED capture EIA (BED-CEIA; named after HIV subtypes B, E, and D), which can be used for detecting recent infections in populations infected by multiple HIV-1 subtypes. The mean BED window period is 156 days. Using the BED assay, Karon et al. [76] further proposed a method which can take into account information on history of HIV testing. Here, testing history refers both to whether an individual has undertaken HIV testing prior to HIV infection as well as the testing frequency. Since the antibody avidity index is always low during early infection, another method for estimation of recent infections based on the avidity index was proposed [77]. Genetic diversity of HIV has also been used as a biomarker to estimate HIV incidence [78–81], since it changes as the disease progresses. Other published studies [78, 79] identified recent HIV-1 infections based on data from traditional or next-generation DNA sequencing. Another research team [80, 81] developed a method based on a high-resolution melting (HRM) diversity assay to determine HIV diversity without sequencing.

### Multiassay algorithms (MAAs)

**Fig. 2:**
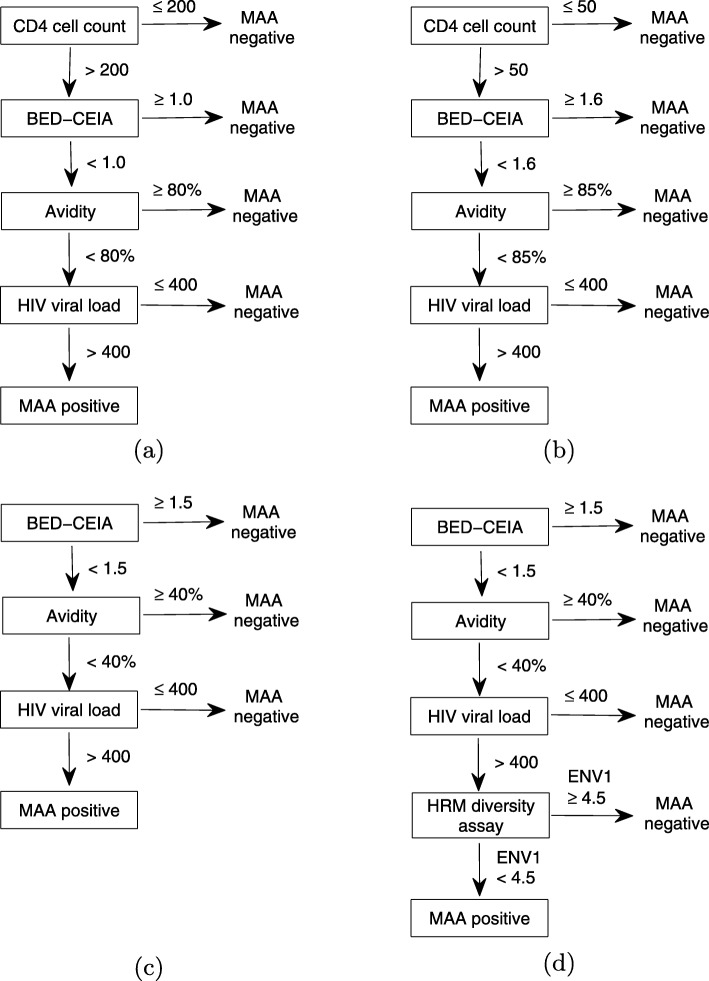
Multi-assay algorithms (MAAs) for cross-sectional HIV incidence estimation (**a**) and (**b**) MAAs using CD4+ T-cell counts with different cut-off values. (**c**) MAA using only three biomarkers. (**d**) MAA using HRM diversity assay

The above serological assays have limitations because of their low accuracy in distinguishing recent from chronic infections. Some chronic infections may be misclassified as recent infection, and thus these methods may overestimate HIV incidence [82, 83]. Laeyendecker et al. [82, 83] demonstrated that factors such as low viral loads, low CD4+ T-cell counts, and >2 years of ART were associated with misclassification by the BED-CEIA. Avidity assays, which identify recent infections by studying the maturity of the antibody response against HIV, also have difficulties in distinguishing recent infections for HIV-1 incidence estimation [87, 88]. Laeyendecker et al. [84] and Brookmeyer [85] developed a MAA to estimate HIV incidence. The MAA integrates data from BED-CEIAs, antibody avidity assays, HIV viral loads and CD4+ T-cell counts. These algorithms are described in Fig. 2a and b, respectively.

All biomarker approaches estimate incidence at a time prior to sample collection, and the concept of the shadow describes the lag-time [85, 89–91]. Shadow and mean window period are two distinct but important concepts for evaluating the statistical accuracy of current HIV incidence estimates. Estimation approaches with large mean window periods will have smaller standard errors, and those with small shadows can better estimate more recent incidence [85, 89–91]. Thus, estimation approaches involving a larger mean window period and a smaller shadow are desirable.

The difference between the MAAs proposed by Laeyendecker et al. [84] and Brookmeyer [85] is that they use different cut-offs for CD4+ T-cell counts, BED-CEIAs, avidity and viral loads. Thus, the two algorithms have different mean window periods (141 days, 95% confidence interval (CI) (94, 150) vs. 159 days, 95% CI (134, 186), respectively) and shadows (128 days vs. 184 days, respectively). As Fig. 2a and b show, both of these algorithms require CD4+ T-cell counts, which are difficult to obtain in some settings. Thus, Laeyendecker et al. [85] developed another MAA using only three biomarkers (BED, avidity, and viral load) as shown in Fig. 2c. This three-biomarker-assay does not require CD4+ T-cell count data, and thus is less expensive. However, the mean window period for the three-biomarker-assay is 58 days shorter than that of the four-biomarker-assay. Hence, to achieve the same incidence standard error, the three-biomarker-assay requires a sample size about 57% larger.

Cousins et al. [86] proposed a new MAA in which a HRM diversity assay is used in place of CD4+ T-cell count data, as shown in Fig. 2d. The mean window period and shadow for the HRM-based MAA are 154 days (95% CI 128, 180 days) and 179 days (95% CI 135, 243 days), respectively. The performance of the HRM-based MAA was shown to be nearly identical to that of the MAA including CD4+ T-cell count data.

For all MAAs, HIV incidence is calculated using the following equation:

$$I=\frac{n}{N}\cdot\frac{1}{T},$$

where *n* is the number of MAA-positive subjects, *N* is the total number of individuals who are HIV seronegative, and *T* is the mean window period.

Several narrative reviews have been published describing incidence estimation approaches that use biomarker data [10, 11, 74, 88, 91–93]. Technical challenges of biomarker approach include misclassification of chronic infections as recent infections and a large variation in testing results between individuals [10], although the accuracy of recent infection estimate has been markedly improved by using MAAs. As reviewed by Murphy et al. [93], biomarker-based incidence could achieve high precision if false recency ratio is sufficiently close to zero. Moreover, as the biomarker approaches include a variety of biomarkers, the complexity to identify recent infections has become more and more complex over time, which may sometimes even require specialized equipments. Early treatment and the use of pre- and post-exposure prophylaxis also bring new challenges to the biomarker approaches. In recent years, the incidence in some populations or sub-populations have been estimated by using biomarker approaches [94–96], and sometimes the biomarker method was combined with other existing modelling approaches [97, 98]. Because of financial constraints, insufficient coordinated action among funding bodies, governments and developers could also act as a hazard for propagating this approach [93], frequently involving problems in purchasing agreement and limited financial support for quality control and training.

## Discussion

In this review, six major methods for estimating HIV incidence were briefly described. These included the back-calculation method, methods using CD4+ T-cell depletion models, methods using HIV case reporting data, methods based on cohort studies, methods using prevalence data, and biomarker-based approaches. Back-calculation methods can be divided into three subgroups according to the data used: (i) AIDS diagnosis data only, (ii) both HIV and AIDS diagnosis data, and (iii) HIV/AIDS diagnosis data as well as CD4+ T-cell counts at diagnosis. Similarly, methods using prevalence data can be further divided into methods based on serial and cross-sectional data. Our primary foci were the background mechanism of estimation, the required data types, the scope of application, the model formulation, the derivation of the maximum likelihood function, and the advantages and disadvantages of applying each method in practice.

Back-calculation methods are widely used to estimate the incidence and prevalence of HIV in various parts of the world [43, 46]. These methods were initially developed using AIDS diagnosis data only, but were later extended to use both HIV and AIDS diagnoses, and then to further account for CD4+ T-cell counts at diagnosis. The back-calculation method has also been modified to include the effect of ART on the distribution of the incubation period. Back-calculation methods have clear advantages and disadvantages compared with other methods [99]. First, the back-calculation method requires only data from case reporting systems, and does not necessarily require laboratory testing and individual-level data. However, the incidence estimate in recent years tends to be unstable, especially where the HIV epidemic has just started, and accuracy of the estimate is influenced by the distribution of the incubation period (or the progression rate) as well as the testing rate.

Compared with back-calculation method, it would be easier to implement the statistical estimation using CD4+ T-cell depletion among non-experts. However, it assumes that the distribution of delays in diagnosis does not change over time. Thus, it may overestimate HIV incidence if HIV testing rates increase over time. As mentioned above, cohort studies have many difficulties and may introduce some biases when incidence is directly estimated among high-risk populations with close follow-up. For methods using prevalence data, both methods using serial and cross-sectional prevalence data are associated with uncertainties in evaluating HIV prevalence and AIDS deaths. Moreover, both methods are strongly influenced by the use of ART. Methods using cross-sectional prevalence data further assume that HIV incidence during the time interval between two prevalence surveys is constant, which is only true for very short time intervals. Biomarker-based approaches, which uses biomarkers in biological samples collected in cross-sectional studies to identify recent HIV infections, can avoid the difficulties associated with follow-up of high-risk uninfected persons in cohort studies as well as difficulties in estimating the distribution of long incubation periods. Biomarker-based methods can better estimate more recent HIV incidence compared with the back-calculation method. As laboratory testing techniques progress, MAAs have become available at low cost, which could minimize the effort and cost involved in incidence estimation in the future. Nevertheless, minimizing the ‘false recency ratio’ (FRR) at a sufficiently low level remains to be a challenge. Biomarker approaches also involve other technical difficulties in quality control, training and evaluation of assays.

The required data are, at the moment, divided into four different categories: (i) epidemiological data including AIDS diagnoses and HIV diagnoses, (ii) CD4 T-cell counts at diagnosis, (iii) prevalence data, and (iv) biomarker testing data. Prevalence data may be further divided into serial prevalence and cross-sectional prevalence data. It must be noted that definitions of HIV incidence are not uniform across different methods. For the back-calculation method, methods using CD4+ T-cell depletion models, methods using cohort studies and methods using serial prevalence data, HIV incidence is defined as the number of new HIV infections per unit time (year) or the instantaneous incident infections occurring at time *t*. However, for methods using cross-sectional prevalence data, HIV incidence is defined as the average hazard of new infections occurring during the interval. For the biomarker approach, an HIV incidence rate is estimated, which is defined as the infection risk per unit time for each uninfected individual (except for the method using BED-CEIA [76], which estimates conventional incidence instead). Obviously, conventional incidence and incidence rates can be converted as long as the total number of uninfected individuals is known. In addition to different incidence definitions, there is also another difference among these methods. The back-calculation method, methods using CD4+ T-cell depletion model, methods using cohort studies and methods using serial prevalence data can estimate serial incidence (i.e., the incidence year-over-year). However, the method using cross-sectional prevalence data and the biomarker approach estimate the cross-sectional incidence or the HIV incidence at a time prior to collection of samples. Thus, different methods estimate HIV incidence with variable time frames.

## Conclusion

A variety of methods exist to estimate HIV incidence from different data types and scopes, and it is difficult to conclude which method perform best. Rather, it should be remembered that HIV incidence estimation itself described what cannot be directly validated, as the estimated quantity is not directly observable in natural settings. Thus, a new method should be regarded as way to mitigate uncertainty with respect to the estimates of another method, and analyzing HIV data from multiple standpoints and sources is one way to overcome such uncertainty. As the methods for HIV incidence estimation have different scopes and different advantages and disadvantages, we hope that this review will be useful for determining which datasets need to be collected to estimate HIV incidence in a comprehensive manner. Should a surveillance system be improved to collect multiple types of datasets as described above, it would be feasible to cross-validate different methodologies and see how different methods can complement each other so that an objective assessment of the HIV/AIDS epidemic will be eventually achieved.

## Data Availability

Not applicable.

## Abbreviations

AIDS: Acquired immunodeficiency syndrome
ART: Antiretroviral therapy
CI: Confidence interval
HIV: Human immunodeficiency virus
UNAIDS: Joint united nations programme on HIV/AIDS
